# First record of *Epeurysadistincta* Huang & Ding (Hemiptera, Delphacidae, Delphacinae) from South Korea, with an illustrated key to the Korean Tropidocephalini species

**DOI:** 10.3897/BDJ.12.e134165

**Published:** 2024-11-06

**Authors:** Sanghyo Park, Wonhoon Lee

**Affiliations:** 1 Department of Plant Medicine and Institute of Agriculture and Life Sciences, Gyeongsang National University, jinju, Republic of Korea Department of Plant Medicine and Institute of Agriculture and Life Sciences, Gyeongsang National University jinju Republic of Korea

**Keywords:** Delphacidae, Tropidocephalini, new record, South Korea

## Abstract

**Background:**

The tribe Tropidocephalini is the second largest tribe of the subfamily Delphacinae, comprising 204 species of 37 genera worldwide. Most species in this tribe feed on bamboo (Bambusoideae) or grasses (Poaceae). In Korea, only three species have been reported.

**New information:**

This tribe is represented by four species in South Korea, including a newly-recorded species, *Epeurysadistincta* Huang & Ding, 1979. Descriptions and illustrations of the four species and a revised key for the identification of the tribe Tropidocephalini are provided.

## Introduction

The tribe Tropidocephalini is the second largest tribe of the subfamily Delphacinae, comprising 207 species of 38 genera worldwide (Bourgoin 2023, Li et al. 2023a, Li et al. 2023b). Most species in this tribe feed on bamboo (Bambusoideae) or other grasses (Poaceae) (Chen and Tsai 2009). This tribe has a broad distribution and they are particularly common in China comprising about 118 species in 24 genera (Li et al. 2023a, Li et al. 2023b). Compared to other tribes of subfamily Delphacinae, the Tropidocephalini has different morphological characteristics: the hind tibial spur is solid, without teeth on the hind margin and the inner surface is slightly concave. Additionally, the symmetrical aedeagus is twisted with a slender process arising from the base.

In Korea, three species of two genera have been recorded in the tribe Tropidocephalini: *Epeurysanawaii* Matsumura, 1900, *Tropidocephalabrunnipennis* (Distant, 1906) and *Tropidocephalanigra* (Matsumura, 1900). In this study, we report *Epeurysadistincta* Huang & Ding, 1979 for the first time from South Korea. We provide morphological characteristics, photographs and taxonomic keys of the four species.

## Materials and methods

Samples of planthoppers were collected from 2021 to 2023 by using two methods, sweeping and light trap, the latter using the Lepiled Maxi UV light trap (Brehm 2017). Samples were preserved on –20℃ refrigerator or pinning.

Images and measurements were taken by LEICA M205C (© Leica Microsystems, Wetzlar, HESSE, Germany). Images were stacked using the software Delta Bio Combine. To examine male genitalia, the male abdomen was soaked in 10% potassium hydroxide (KOH) and boiled in a heating block (70℃) for 90 minutes. After that, separated genitalia were observed under a microscope (LEICA M205C and TUCSEN Dhyana 400DC) with glycerine. All specimens were deposited in Institute of Agriculture & Life Science, Gyeongsang National University.

## Taxon treatments

### Epeurysa distincta

Distant, 1912

D0B7962B-3CD1-5299-905F-EEEA72262D23

*Epeurysadistincta* Huang & Ding, 1979: 178
*Epeurysainfumata* Yang & Yang, 1986: 47

#### Materials

- **Type status:** Other material. **Occurrence:** recordedBy: Sanghyo Park; individualCount: 1; sex: male; lifeStage: adult; occurrenceID: 3221F525-BC8F-5988-B92E-23704F586070; **Taxon:** scientificName: Epeurysadistincta; kingdom: Animal; phylum: Arthropoda; class: Insecta; order: Hemiptera; family: Delphacidae; genus: Epeurysa; **Location:** country: South Korea; stateProvince: Jeju-do; municipality: Seogwipo-si; locality: 791, Seohong-dong; georeferenceProtocol: label; **Identification:** identifiedBy: Sanghyo Park; dateIdentified: 2023; **Event:** eventDate: 07-05-2023; **Record Level:** language: en; collectionCode: Insects; basisOfRecord: PreservedSpecimen
- **Type status:** Other material. **Occurrence:** recordedBy: Sanghyo Park; individualCount: 2; sex: male; lifeStage: adult; occurrenceID: D4E3398D-0BA2-5D69-9514-2EEB0D4E5524; **Taxon:** scientificName: Epeurysadistincta; kingdom: Animal; phylum: Arthropoda; class: Insecta; order: Hemiptera; family: Delphacidae; genus: Epeurysa; **Location:** country: South Korea; stateProvince: Jeju-do; municipality: Seogwipo-si; locality: 18-2, Topyeong-ro; georeferenceProtocol: label; **Identification:** identifiedBy: Sanghyo Park; dateIdentified: 2023; **Event:** eventDate: 07-05-2023; **Record Level:** language: en; collectionCode: Insects; basisOfRecord: PreservedSpecimen

#### Description

Body length of male 3.8 mm (with tegmina). General colouration brown. Vertex very short, about two times wider than the length, lateral margins more or less divergent apically and also basally, carinae comparatively indistinct. Frons brown, width more than half the frons length, genae and clypeus brown. Pronotum and mesonotum tricarinate. Fore-wings transparent and light brown, the veins brown, distinctly tinged two dark brown spot in clavus and middle of apex portion; abdominal segments dark brown (Figs 1a, 2a).

**Male genitalia.** Pygofer much longer ventrally than dorsally, laterodorsal angles not produced ventrally, medioventral process two times longer than lateroventral processes, apically ovoid. Genital stylet strongly outwardly curved and divided into two branches in the middle. Anal segment with two small processes. Aedeagus with two branched, curved and both narrowing towards the apex (Figs 3a, 4a).

**Measurements.** Male macropterous form (n = 3). Body length without tegmina: 2.46 mm; body length with tegmina: 3.84 mm; body width: 0.94 mm; head length: 0.34 mm; head width (including eyes): 0.74 mm; 1^st^ antennal segment length: 0.06 mm; 2^nd^ antennal segment length: 0.16 mm; vertex length: 0.19 mm; vertex width: 0.34 mm; frons length: 0.44 mm; frons width: 0.35 mm; pronotum length: 0.24 mm; pronotum width: 0.76 mm; mesonotum length: 0.64 mm; mesonotum width: 0.70 mm.

#### Diagnosis

This species is very similar to *Epeurysanawaii*. However, it is distinguished by the strongly outwardly curved genital stylet and the brown fore-wing veins with darker sockets for the setae (Fig. 5).

#### Distribution

Korea (new record), Japan (Fujinuma 2016), China (Hunan, Taiwan, Guizhou, Yunnan) (Ding 2006).

#### Host plants

Phyllostachysnigravar.henonis Stapf ex Rendle. (Poaceae) (in this study).

### Epeurysa nawaii

Distant, 1912

7EC4D89A-3F39-54BD-A030-5E97ED774BCE

*Epeurysanawaii* Matsumura, 1900: 261
*Eurysanawaii* Choe 1981: 21

#### Materials

- **Type status:** Other material. **Occurrence:** recordedBy: Sanghyo Park; individualCount: 1; sex: male; lifeStage: adult; occurrenceID: 6A832EF2-9802-5318-87B7-8A39EA4C84D3; **Taxon:** scientificName: Epeurysanawaii; kingdom: Animal; phylum: Arthropoda; class: Insecta; order: Hemiptera; family: Delphacidae; genus: Epeurysa; **Location:** country: South Korea; stateProvince: Gyeongsangnam-do; municipality: Jinju-si; locality: 1300-4, Gajwa-dong; georeferenceProtocol: label; **Identification:** identifiedBy: Sanghyo Park; dateIdentified: 2021; **Event:** eventDate: 07-29-2021; **Record Level:** language: en; collectionCode: Insects; basisOfRecord: PreservedSpecimen
- **Type status:** Other material. **Occurrence:** recordedBy: Sanghyo Park; individualCount: 4; sex: male; lifeStage: adult; occurrenceID: 486C58D0-5244-57A4-93E8-A41266434050; **Taxon:** scientificName: Epeurysanawaii; kingdom: Animal; phylum: Arthropoda; class: Insecta; order: Hemiptera; family: Delphacidae; genus: Epeurysa; **Location:** country: South Korea; stateProvince: Gyeongsangnam-do; municipality: Jinju-si; locality: 20-13, Sanyu-ro 469beon-gil; georeferenceProtocol: label; **Identification:** identifiedBy: Sanghyo Park; dateIdentified: 2022; **Event:** eventDate: 06-25-2022; **Record Level:** language: en; collectionCode: Insects; basisOfRecord: PreservedSpecimen
- **Type status:** Other material. **Occurrence:** recordedBy: Sanghyo Park; individualCount: 1; sex: male; lifeStage: adult; occurrenceID: FAD0D0EE-ACFA-5711-8081-DD8CFBCA4D72; **Taxon:** scientificName: Epeurysanawaii; kingdom: Animal; phylum: Arthropoda; class: Insecta; order: Hemiptera; family: Delphacidae; genus: Epeurysa; **Location:** country: South Korea; stateProvince: Gyeongsangnam-do; municipality: Jinju-si; locality: 815, Gawja-dong; georeferenceProtocol: label; **Identification:** identifiedBy: Sanghyo Park; dateIdentified: 2023; **Event:** eventDate: 06-02-2022; **Record Level:** language: en; collectionCode: Insects; basisOfRecord: PreservedSpecimen
- **Type status:** Other material. **Occurrence:** recordedBy: Sanghyo Park; individualCount: 7; sex: female; lifeStage: adult; occurrenceID: 25E80AE4-EB95-558B-AF6D-06ED98576AB2; **Taxon:** scientificName: Epeurysanawaii; kingdom: Animal; phylum: Arthropoda; class: Insecta; order: Hemiptera; family: Delphacidae; genus: Epeurysa; **Location:** country: South Korea; stateProvince: Gyeongsangnam-do; municipality: Jinju-si; locality: 815, Gawja-dong; georeferenceProtocol: label; **Identification:** identifiedBy: Sanghyo Park; dateIdentified: 2023; **Event:** eventDate: 06-02-2022; **Record Level:** language: en; collectionCode: Insects; basisOfRecord: PreservedSpecimen
- **Type status:** Other material. **Occurrence:** recordedBy: Sanghyo Park; individualCount: 5; sex: male; lifeStage: adult; occurrenceID: 4605BC88-0DCD-5848-B782-D0F03D386EDF; **Taxon:** scientificName: Epeurysanawaii; kingdom: Animal; phylum: Arthropoda; class: Insecta; order: Hemiptera; family: Delphacidae; genus: Epeurysa; **Location:** country: South Korea; stateProvince: Gyeongsangnam-do; municipality: Jinju-si; locality: 1282-40, Indam-ri Geumgok-myeon; georeferenceProtocol: label; **Identification:** identifiedBy: Sanghyo Park; dateIdentified: 2023; **Event:** eventDate: 07-25-2023; **Record Level:** language: en; collectionCode: Insects; basisOfRecord: PreservedSpecimen
- **Type status:** Other material. **Occurrence:** recordedBy: Sanghyo Park; individualCount: 5; sex: female; lifeStage: adult; occurrenceID: F601FF62-B7D4-5CDF-9DE7-7892E53AEE23; **Taxon:** scientificName: Epeurysanawaii; kingdom: Animal; phylum: Arthropoda; class: Insecta; order: Hemiptera; family: Delphacidae; genus: Epeurysa; **Location:** country: South Korea; stateProvince: Gyeongsangnam-do; municipality: Jinju-si; locality: 1282-40, Indam-ri Geumgok-myeon; georeferenceProtocol: label; **Identification:** identifiedBy: Sanghyo Park; dateIdentified: 2023; **Event:** eventDate: 07-25-2023; **Record Level:** language: en; collectionCode: Insects; basisOfRecord: PreservedSpecimen
- **Type status:** Other material. **Occurrence:** recordedBy: Sanghyo Park; individualCount: 1; sex: male; lifeStage: adult; occurrenceID: 3A672DAC-951B-5F0D-A7F7-C5F632548D15; **Taxon:** scientificName: Epeurysanawaii; kingdom: Animal; phylum: Arthropoda; class: Insecta; order: Hemiptera; family: Delphacidae; genus: Epeurysa; **Location:** country: South Korea; stateProvince: Gyeongsangbuk-do; municipality: Yeongdeok-gun; locality: 31, Gisa-ri Jipum-myeon; georeferenceProtocol: label; **Identification:** identifiedBy: Sanghyo Park; dateIdentified: 2023; **Event:** eventDate: 06-17-2023; **Record Level:** language: en; collectionCode: Insects; basisOfRecord: PreservedSpecimen
- **Type status:** Other material. **Occurrence:** recordedBy: Sanghyo Park; individualCount: 4; sex: male; lifeStage: adult; occurrenceID: DD6583DD-6CBB-5139-9865-D9EF413A4AF6; **Taxon:** scientificName: Epeurysanawaii; kingdom: Animal; phylum: Arthropoda; class: Insecta; order: Hemiptera; family: Delphacidae; genus: Epeurysa; **Location:** country: South Korea; stateProvince: Jeollabuk-do; municipality: Wanju-gun; locality: 591-3, Geumpyeong-ri Iseo-myeon; georeferenceProtocol: label; **Identification:** identifiedBy: Sanghyo Park; dateIdentified: 2022; **Event:** eventDate: 06-11-2022; **Record Level:** language: en; collectionCode: Insects; basisOfRecord: PreservedSpecimen
- **Type status:** Other material. **Occurrence:** recordedBy: Sanghyo Park; individualCount: 1; sex: male; lifeStage: adult; occurrenceID: A3FF8A09-F98F-5011-9EA6-5BEDE8A7661B; **Taxon:** scientificName: Epeurysanawaii; kingdom: Animal; phylum: Arthropoda; class: Insecta; order: Hemiptera; family: Delphacidae; genus: Epeurysa; **Location:** country: South Korea; stateProvince: Jeollabuk-do; municipality: Jeonju-si; locality: 462-45, Beonyeong-ro Deokjin-gu; georeferenceProtocol: label; **Identification:** identifiedBy: Sanghyo Park; dateIdentified: 2022; **Event:** eventDate: 06-12-2022; **Record Level:** language: en; collectionCode: Insects; basisOfRecord: PreservedSpecimen
- **Type status:** Other material. **Occurrence:** recordedBy: Sanghyo Park; individualCount: 1; sex: male; lifeStage: adult; occurrenceID: B409658A-E0A0-5327-A490-9CAAE4144F90; **Taxon:** scientificName: Epeurysanawaii; kingdom: Animal; phylum: Arthropoda; class: Insecta; order: Hemiptera; family: Delphacidae; genus: Epeurysa; **Location:** country: South Korea; stateProvince: Jeollanam-do; municipality: Sinan-gun; locality: 40, Jangdo-gil, Heuksan-myeon; georeferenceProtocol: label; **Identification:** identifiedBy: Sanghyo Park; dateIdentified: 2022; **Event:** eventDate: 05-18-2022; **Record Level:** language: en; collectionCode: Insects; basisOfRecord: PreservedSpecimen
- **Type status:** Other material. **Occurrence:** recordedBy: Sanghyo Park; individualCount: 1; sex: male; lifeStage: adult; occurrenceID: 960F8559-F386-56F7-A08C-C6606FF58C41; **Taxon:** scientificName: Epeurysanawaii; kingdom: Animal; phylum: Arthropoda; class: Insecta; order: Hemiptera; family: Delphacidae; genus: Epeurysa; **Location:** country: South Korea; stateProvince: Jeollanam-do; municipality: Suncheon-si; locality: 295-8, Jogok-dong; georeferenceProtocol: label; **Identification:** identifiedBy: Sanghyo Park; dateIdentified: 2023; **Event:** eventDate: 03-29-2023; **Record Level:** language: en; collectionCode: Insects; basisOfRecord: PreservedSpecimen
- **Type status:** Other material. **Occurrence:** recordedBy: Sanghyo Park; individualCount: 2; sex: female; lifeStage: adult; occurrenceID: 21A80050-24DF-585D-9D6C-2E15A04CB3DD; **Taxon:** scientificName: Epeurysanawaii; kingdom: Animal; phylum: Arthropoda; class: Insecta; order: Hemiptera; family: Delphacidae; genus: Epeurysa; **Location:** country: South Korea; stateProvince: Jeollanam-do; municipality: Suncheon-si; locality: 295-9, Jogok-dong; georeferenceProtocol: label; **Identification:** identifiedBy: Sanghyo Park; dateIdentified: 2023; **Event:** eventDate: 03-29-2023; **Record Level:** language: en; collectionCode: Insects; basisOfRecord: PreservedSpecimen
- **Type status:** Other material. **Occurrence:** recordedBy: Sanghyo Park; individualCount: 1; sex: male; lifeStage: adult; occurrenceID: 873B4724-8EFF-5EC0-9AA5-1D3C1D6BCF3A; **Taxon:** scientificName: Epeurysanawaii; kingdom: Animal; phylum: Arthropoda; class: Insecta; order: Hemiptera; family: Delphacidae; genus: Epeurysa; **Location:** country: South Korea; stateProvince: Jeju-do; municipality: Seogwipo-si; locality: 2175, Donghong-dong; georeferenceProtocol: label; **Identification:** identifiedBy: Sanghyo Park; dateIdentified: 2023; **Event:** eventDate: 07-08-2023; **Record Level:** language: en; collectionCode: Insects; basisOfRecord: PreservedSpecimen
- **Type status:** Other material. **Occurrence:** recordedBy: Sanghyo Park; individualCount: 2; sex: female; lifeStage: adult; occurrenceID: B8E08661-1D31-5CE6-AF24-DDC6D1E60819; **Taxon:** scientificName: Epeurysanawaii; kingdom: Animal; phylum: Arthropoda; class: Insecta; order: Hemiptera; family: Delphacidae; genus: Epeurysa; **Location:** country: South Korea; stateProvince: Jeju-do; municipality: Seogwipo-si; locality: 2176, Donghong-dong; georeferenceProtocol: label; **Identification:** identifiedBy: Sanghyo Park; dateIdentified: 2023; **Event:** eventDate: 07-08-2023; **Record Level:** language: en; collectionCode: Insects; basisOfRecord: PreservedSpecimen

#### Description

Body length of male 3.8 mm. General colouration brown, dark form with wings darker at apical half. Vertex relatively short, base is longer than median length by about three times. Frons relatively wide and brown, frons at mid-line longer than wide, of widest part about 1.3:1. Genae and clypeus also brown. Pronotum and mesonotum tricarinate and brown. Fore-wings generally brown, sometimes darker brown tinges on wings. Abdominal segments dark brown (Figs 1b, 2b).

**Male genitalia.** Pygofer much longer ventrally than dorsally, laterodorsal angles not produced ventrally, medioventral process three times longer than lateral processes, apically ovoid. Genital stylet moderately long, with basal angles very strongly produced to mediocaudad, in caudal view about half as high as inner angle. Anal segment with spinal process, each developed as a convex triangular lobe. Aedeagus with two branched, one branch narrowing towards the apex, another one wider towards the apex (Figs 3b, 4b, Ding 2006).

**Measurements.** Male macropterous form (n = 3). Body length without tegmina: 2.40 mm; body length with tegmina: 3.84 mm; body width: 0.95 mm; head length: 0.40 mm; head width (including eyes): 0.77 mm; 1^st^ antennal segment length: 0.08 mm; 2^nd^ antennal segment length: 0.16 mm; vertex length: 0.16 mm; vertex width: 0.44 mm; frons length: 0.47 mm; frons width: 0.39 mm; pronotum length: 0.23 mm; pronotum width: 0.81 mm; mesonotum length: 0.69 mm; mesonotum width: 0.73 mm.

#### Distribution

Korea, China (Shaanxi, Gansu, Jiangsu, Anhui, Zhejiang, Jiangxi, Hunan, Hubei, Fujinan, Taiwan, Guangdong, Guangxi, Hainan, Sichuan, Guizhou, Yunnan), Japan, Russia (Primorsky Region), Japan, Sri Lanka (Ding 2006).

#### Host plants

*Imperatacylindrica* (Linn.) Raeusch (Poaceae) (in this study), *Phyllostachysbambusoides* Sieb et Zucc (Poaceae) (Ding 2006).

### Tropidocephala brunnipennis

Signoret, 1860

59D1F80A-9559-53F0-8F0B-8FD8BE647BCF

*Tropidocephalabrunnipennis* Signoret, 1860: 185

#### Materials

- **Type status:** Other material. **Occurrence:** recordedBy: Sanghyo Park; individualCount: 11; sex: male; lifeStage: adult; occurrenceID: 7C6566B9-0277-51B1-BB0F-6AFC7ACF6CA4; **Taxon:** scientificName: Tropidocephalabrunnipennis; kingdom: Animal; phylum: Arthropoda; class: Insecta; order: Hemiptera; family: Delphacidae; genus: Tropidocephala; **Location:** country: South Korea; stateProvince: Gyeongsangnam-do; municipality: Tongyeong-si; locality: 60, Inpyeong-dong; georeferenceProtocol: label; **Identification:** identifiedBy: Sanghyo Park; dateIdentified: 2022; **Event:** eventDate: 04-07-2022; **Record Level:** language: en; collectionCode: Insects; basisOfRecord: PreservedSpecimen
- **Type status:** Other material. **Occurrence:** recordedBy: Sanghyo Park; individualCount: 9; sex: female; lifeStage: adult; occurrenceID: 07E3F013-02BB-56E4-8738-64F156754F21; **Taxon:** scientificName: Tropidocephalabrunnipennis; kingdom: Animal; phylum: Arthropoda; class: Insecta; order: Hemiptera; family: Delphacidae; genus: Tropidocephala; **Location:** country: South Korea; stateProvince: Gyeongsangnam-do; municipality: Tongyeong-si; locality: 60, Inpyeong-dong; georeferenceProtocol: label; **Identification:** identifiedBy: Sanghyo Park; dateIdentified: 2022; **Event:** eventDate: 04-07-2022; **Record Level:** language: en; collectionCode: Insects; basisOfRecord: PreservedSpecimen
- **Type status:** Other material. **Occurrence:** recordedBy: Sanghyo Park; individualCount: 11; sex: male; lifeStage: adult; occurrenceID: 2E627D89-BF33-50A7-A368-70FA70AB52E0; **Taxon:** scientificName: Tropidocephalabrunnipennis; kingdom: Animal; phylum: Arthropoda; class: Insecta; order: Hemiptera; family: Delphacidae; genus: Tropidocephala; **Location:** country: South Korea; stateProvince: Gyeongsangnam-do; municipality: Geoje-si; locality: San50-2, Galgot-ri; georeferenceProtocol: label; **Identification:** identifiedBy: Sanghyo Park; dateIdentified: 2023; **Event:** eventDate: 07-30-2023; **Record Level:** language: en; collectionCode: Insects; basisOfRecord: PreservedSpecimen
- **Type status:** Other material. **Occurrence:** recordedBy: Sanghyo Park; individualCount: 15; sex: female; lifeStage: adult; occurrenceID: BFD13F57-C93B-504B-89B7-5412E5EB1F37; **Taxon:** scientificName: Tropidocephalabrunnipennis; kingdom: Animal; phylum: Arthropoda; class: Insecta; order: Hemiptera; family: Delphacidae; genus: Tropidocephala; **Location:** country: South Korea; stateProvince: Gyeongsangnam-do; municipality: Geoje-si; locality: San50-2, Galgot-ri; georeferenceProtocol: label; **Identification:** identifiedBy: Sanghyo Park; dateIdentified: 2023; **Event:** eventDate: 07-30-2023; **Record Level:** language: en; collectionCode: Insects; basisOfRecord: PreservedSpecimen
- **Type status:** Other material. **Occurrence:** recordedBy: Sanghyo Park; individualCount: 2; sex: male; lifeStage: adult; occurrenceID: B181BD52-5D4A-5534-A01C-65082983BA5A; **Taxon:** scientificName: Tropidocephalabrunnipennis; kingdom: Animal; phylum: Arthropoda; class: Insecta; order: Hemiptera; family: Delphacidae; genus: Tropidocephala; **Location:** country: South Korea; stateProvince: Gyeongsangnam-do; municipality: Namhae-gun; locality: 97 Sports-ro 287beon-gil, Namhae-eup; georeferenceProtocol: label; **Identification:** identifiedBy: Sanghyo Park; dateIdentified: 2023; **Event:** eventDate: 04-13-2023; **Record Level:** language: en; collectionCode: Insects; basisOfRecord: PreservedSpecimen
- **Type status:** Other material. **Occurrence:** recordedBy: Sanghyo Park; individualCount: 1; sex: female; lifeStage: adult; occurrenceID: FE240F88-49BF-5F8C-97B1-E208F796E3F8; **Taxon:** scientificName: Tropidocephalabrunnipennis; kingdom: Animal; phylum: Arthropoda; class: Insecta; order: Hemiptera; family: Delphacidae; genus: Tropidocephala; **Location:** country: South Korea; stateProvince: Gyeongsangnam-do; municipality: Namhae-gun; locality: 97 Sports-ro 287beon-gil, Namhae-eup; georeferenceProtocol: label; **Identification:** identifiedBy: Sanghyo Park; dateIdentified: 2023; **Event:** eventDate: 04-13-2023; **Record Level:** language: en; collectionCode: Insects; basisOfRecord: PreservedSpecimen
- **Type status:** Other material. **Occurrence:** recordedBy: Sanghyo Park; individualCount: 3; sex: male; lifeStage: adult; occurrenceID: 53588582-B5FD-59A8-91CB-24B4752D5B0A; **Taxon:** scientificName: Tropidocephalabrunnipennis; kingdom: Animal; phylum: Arthropoda; class: Insecta; order: Hemiptera; family: Delphacidae; genus: Tropidocephala; **Location:** country: South Korea; stateProvince: Jeollanam-do; municipality: Sinan-gun; locality: 543, Ye-ri, Heuksan-myeon; georeferenceProtocol: label; **Identification:** identifiedBy: Sanghyo Park; dateIdentified: 2022; **Event:** eventDate: 06-18-2022; **Record Level:** language: en; collectionCode: Insects; basisOfRecord: PreservedSpecimen
- **Type status:** Other material. **Occurrence:** recordedBy: Sanghyo Park; individualCount: 3; sex: male; lifeStage: adult; occurrenceID: EC731580-F6CD-5385-AB01-9EE707E31CC8; **Taxon:** scientificName: Tropidocephalabrunnipennis; kingdom: Animal; phylum: Arthropoda; class: Insecta; order: Hemiptera; family: Delphacidae; genus: Tropidocephala; **Location:** country: South Korea; stateProvince: Jeollanam-do; municipality: Suncheon-si; locality: 13-36, Yulchonsandan 5-ro, Haeryong-myeon; georeferenceProtocol: label; **Identification:** identifiedBy: Sanghyo Park; dateIdentified: 2022; **Event:** eventDate: 07-27-2022; **Record Level:** language: en; collectionCode: Insects; basisOfRecord: PreservedSpecimen
- **Type status:** Other material. **Occurrence:** recordedBy: Sanghyo Park; individualCount: 4; sex: female; lifeStage: adult; occurrenceID: CBF65CAB-A3DD-54CE-8EF1-8E93309651B7; **Taxon:** scientificName: Tropidocephalabrunnipennis; kingdom: Animal; phylum: Arthropoda; class: Insecta; order: Hemiptera; family: Delphacidae; genus: Tropidocephala; **Location:** country: South Korea; stateProvince: Jeollanam-do; municipality: Suncheon-si; locality: 13-36, Yulchonsandan 5-ro, Haeryong-myeon; georeferenceProtocol: label; **Identification:** identifiedBy: Sanghyo Park; dateIdentified: 2022; **Event:** eventDate: 07-27-2022; **Record Level:** language: en; collectionCode: Insects; basisOfRecord: PreservedSpecimen
- **Type status:** Other material. **Occurrence:** recordedBy: Sanghyo Park; individualCount: 4; sex: male; lifeStage: adult; occurrenceID: A8428C97-0225-5E6C-A709-366A4B4EB025; **Taxon:** scientificName: Tropidocephalabrunnipennis; kingdom: Animal; phylum: Arthropoda; class: Insecta; order: Hemiptera; family: Delphacidae; genus: Tropidocephala; **Location:** country: South Korea; stateProvince: Jeollanam-do; municipality: Gwangyang-si; locality: 18-42, Hangman 8-ro; georeferenceProtocol: label; **Identification:** identifiedBy: Sanghyo Park; dateIdentified: 2022; **Event:** eventDate: 07-27-2022; **Record Level:** language: en; collectionCode: Insects; basisOfRecord: PreservedSpecimen
- **Type status:** Other material. **Occurrence:** recordedBy: Sanghyo Park; individualCount: 13; sex: female; lifeStage: adult; occurrenceID: 25E52B4C-FC59-59D4-9BDB-9C6AABCB72B8; **Taxon:** scientificName: Tropidocephalabrunnipennis; kingdom: Animal; phylum: Arthropoda; class: Insecta; order: Hemiptera; family: Delphacidae; genus: Tropidocephala; **Location:** country: South Korea; stateProvince: Jeollanam-do; municipality: Gwangyang-si; locality: 18-42, Hangman 8-ro; georeferenceProtocol: label; **Identification:** identifiedBy: Sanghyo Park; dateIdentified: 2022; **Event:** eventDate: 07-27-2022; **Record Level:** language: en; collectionCode: Insects; basisOfRecord: PreservedSpecimen
- **Type status:** Other material. **Occurrence:** recordedBy: Sanghyo Park; individualCount: 1; sex: male; lifeStage: adult; occurrenceID: 3289636C-5382-5F3A-ADCD-53AE4105F0D1; **Taxon:** scientificName: Tropidocephalabrunnipennis; kingdom: Animal; phylum: Arthropoda; class: Insecta; order: Hemiptera; family: Delphacidae; genus: Tropidocephala; **Location:** country: South Korea; stateProvince: Jeju-do; municipality: Jeju-si; locality: 72, Sumogwon-gil; georeferenceProtocol: label; **Identification:** identifiedBy: Sanghyo Park; dateIdentified: 2023; **Event:** eventDate: 07-03-2023; **Record Level:** language: en; collectionCode: Insects; basisOfRecord: PreservedSpecimen
- **Type status:** Other material. **Occurrence:** recordedBy: Sanghyo Park; individualCount: 2; sex: female; lifeStage: adult; occurrenceID: 4E2F1933-AA4F-5F95-95FF-B3E0197A1349; **Taxon:** scientificName: Tropidocephalabrunnipennis; kingdom: Animal; phylum: Arthropoda; class: Insecta; order: Hemiptera; family: Delphacidae; genus: Tropidocephala ; **Location:** country: South Korea; stateProvince: Jeju-do; municipality: Jeju-si; locality: 72, Sumogwon-gil; georeferenceProtocol: label; **Identification:** identifiedBy: Sanghyo Park; dateIdentified: 2023; **Event:** eventDate: 07-03-2023; **Record Level:** language: en; collectionCode: Insects; basisOfRecord: PreservedSpecimen
- **Type status:** Other material. **Occurrence:** recordedBy: Sanghyo Park; individualCount: 1; sex: male; lifeStage: adult; occurrenceID: 0B8462F8-2E80-5BCD-9B3B-77131229F0F4; **Taxon:** scientificName: Tropidocephalabrunnipennis; kingdom: Animal; phylum: Arthropoda; class: Insecta; order: Hemiptera; family: Delphacidae; genus: Tropidocephala ; **Location:** country: South Korea; stateProvince: Jeju-do; municipality: Jeju-si; locality: 72, Sumogwon-gil; georeferenceProtocol: label; **Identification:** identifiedBy: Sanghyo Park; dateIdentified: 2023; **Event:** eventDate: 07-03-2023; **Record Level:** language: en; collectionCode: Insects; basisOfRecord: PreservedSpecimen
- **Type status:** Other material. **Occurrence:** recordedBy: Sanghyo Park; individualCount: 2; sex: female; lifeStage: adult; occurrenceID: 5E60BBB0-FC96-559E-8FE9-6447F84FDBAD; **Taxon:** scientificName: Tropidocephalabrunnipennis; kingdom: Animal; phylum: Arthropoda; class: Insecta; order: Hemiptera; family: Delphacidae; genus: Tropidocephala; **Location:** country: South Korea; stateProvince: Jeju-do; municipality: Jeju-si; locality: 72, Sumogwon-gil; georeferenceProtocol: label; **Identification:** identifiedBy: Sanghyo Park; dateIdentified: 2023; **Event:** eventDate: 07-03-2023; **Record Level:** language: en; collectionCode: Insects; basisOfRecord: PreservedSpecimen
- **Type status:** Other material. **Occurrence:** recordedBy: Sanghyo Park; individualCount: 4; sex: male; lifeStage: adult; occurrenceID: A83607CB-5888-513B-BE03-00750A032DB6; **Taxon:** scientificName: Tropidocephalabrunnipennis; kingdom: Animal; phylum: Arthropoda; class: Insecta; order: Hemiptera; family: Delphacidae; genus: Tropidocephala; **Location:** country: South Korea; stateProvince: Jeju-do; municipality: Seogwipo-si; locality: 18-2, Topyeong-ro; georeferenceProtocol: label; **Identification:** identifiedBy: Sanghyo Park; dateIdentified: 2023; **Event:** eventDate: 07-05-2023; **Record Level:** language: en; collectionCode: Insects; basisOfRecord: PreservedSpecimen
- **Type status:** Other material. **Occurrence:** recordedBy: Sanghyo Park; individualCount: 7; sex: female; lifeStage: adult; occurrenceID: 32484386-1FD7-596E-BB78-B24832BFFFEF; **Taxon:** scientificName: Tropidocephalabrunnipennis; kingdom: Animal; phylum: Arthropoda; class: Insecta; order: Hemiptera; family: Delphacidae; genus: Tropidocephala; **Location:** country: South Korea; stateProvince: Jeju-do; municipality: Seogwipo-si; locality: 18-2, Topyeong-ro; georeferenceProtocol: label; **Identification:** identifiedBy: Sanghyo Park; dateIdentified: 2023; **Event:** eventDate: 07-05-2023; **Record Level:** language: en; collectionCode: Insects; basisOfRecord: PreservedSpecimen

#### Description

**Description.** Body length of male 3.1 mm. Body colour greenish-brown to dark brown, vertex and mesonotum greenish-yellow. Vertex length about as long as basal width, outer areas from mediolateral carinae pale brown; frons greenish to blackish towards clypeus and genae below eyes black. Clypeus tricarinate and black. Pronotum and mesonotum with pale brown carinae. Fore-wings mostly blackish with white spot on apical part and green spot on clavus part abdominal segments dark brown, pale yellow posteriorly (Figs 1c, 2c).

**Male genitalia.** Pygofer ovoid and posterior view with opening longer than broad. Genital stylet flattened, apical third about twice as broad at base, inner margin short processes towards inside, forked at apex. Anal style surpassing anterior margins of the long anal segment. Aedeagus two branched slender needle-shaped (Figs 3c, 4c).

**Measurements.** Male macropterous form (n = 3). Body length without tegmina: 2.06 mm; body length with tegmina: 3.09 mm; body width: 0.88 mm; head length: 0.47 mm; head width (including eyes): 0.52 mm; 1^st^ antennal segment length: 0.07 mm; 2^nd^ antennal segment length: 0.11 mm; vertex length: 0.30 mm; vertex width: 0.26 mm; frons length: 0.53 mm; frons width: 0.30 mm; pronotum length: 0.27 mm; pronotum width: 058 mm; mesonotum length: 0.61 mm; mesonotum width: 0.59 mm.

#### Distribution

Korea, China (Gansu, Jiangsu, Anhui, Zhejiang, Jiangxi, Hunan, Fujinan, Taiwan, Guangdong, Guangxi, Hainan, Sichunan, Guizhou, Yunnan), Japan, India, Phillippines, Sri Lanka, Indonesia, Malaysia, New Guinea, Australia, Madagascar, North Africa, southern Europe (Ding 2006).

#### Host plants

*Miscanthussinensis* Anderss (Poaceae) (in this study), *Imperatacylindrica* (Linn.) (Poaceae) (Ding 2006).

### Tropidocephala nigra

(Matsumura, 1900)

0CD4096B-77ED-5C79-BCCD-595BA069A4AA

*Conicodanigra* Matsumura, 1900: 261
*Tropidocephalanigra* Matsumura, 1907: 65

#### Materials

- **Type status:** Other material. **Occurrence:** recordedBy: Sanghyo Park; individualCount: 3; sex: male; lifeStage: adult; occurrenceID: A6C438A1-A850-5864-B969-922071EAC9AB; **Taxon:** scientificName: Tropidocephalanigra; kingdom: Animal; phylum: Arthropoda; class: Insecta; order: Hemiptera; family: Delphacidae; genus: Tropidocephala; **Location:** country: South Korea; stateProvince: Gyeongsangnam-do; municipality: Geoje-si; locality: San 48-38, Galgot-ri, Nambu-myeon; georeferenceProtocol: label; **Identification:** identifiedBy: Sanghyo Park; dateIdentified: 2022; **Event:** eventDate: 07-30-2022; **Record Level:** language: en; collectionCode: Insects; basisOfRecord: PreservedSpecimen
- **Type status:** Other material. **Occurrence:** recordedBy: Sanghyo Park; individualCount: 4; sex: female; lifeStage: adult; occurrenceID: 3165CD56-D62D-5D98-9BC6-BB7135302BBA; **Taxon:** scientificName: Tropidocephalanigra; kingdom: Animal; phylum: Arthropoda; class: Insecta; order: Hemiptera; family: Delphacidae; genus: Tropidocephala; **Location:** country: South Korea; stateProvince: Gyeongsangnam-do; municipality: Geoje-si; locality: San 48-38, Galgot-ri, Nambu-myeon; georeferenceProtocol: label; **Identification:** identifiedBy: Sanghyo Park; dateIdentified: 2022; **Event:** eventDate: 07-30-2022; **Record Level:** language: en; collectionCode: Insects; basisOfRecord: PreservedSpecimen
- **Type status:** Other material. **Occurrence:** recordedBy: Sanghyo Park; individualCount: 3; sex: female; lifeStage: adult; occurrenceID: FDF1CF25-9D8F-54A9-B911-96DCA1076415; **Taxon:** scientificName: Tropidocephalanigra; kingdom: Animal; phylum: Arthropoda; class: Insecta; order: Hemiptera; family: Delphacidae; genus: Tropidocephala; **Location:** country: South Korea; stateProvince: Gyeongsangnam-do; municipality: Geoje-si; locality: 195-1, Gucheon-ri, Dongbu-myeon; georeferenceProtocol: label; **Identification:** identifiedBy: Sanghyo Park; dateIdentified: 2022; **Event:** eventDate: 07-30-2022; **Record Level:** language: en; collectionCode: Insects; basisOfRecord: PreservedSpecimen
- **Type status:** Other material. **Occurrence:** recordedBy: Sanghyo Park; individualCount: 2; sex: male; lifeStage: adult; occurrenceID: 28EA0DD6-1A5F-52F8-9F98-5395EE70F1F1; **Taxon:** scientificName: Tropidocephalanigra; kingdom: Animal; phylum: Arthropoda; class: Insecta; order: Hemiptera; family: Delphacidae; genus: Tropidocephala; **Location:** country: South Korea; stateProvince: Gyeongsangnam-do; municipality: Namhae-gun; locality: 97, Sports-ro 287beon-gil, Namhae-eup; georeferenceProtocol: label; **Identification:** identifiedBy: Sanghyo Park; dateIdentified: 2023; **Event:** eventDate: 04-13-2023; **Record Level:** language: en; collectionCode: Insects; basisOfRecord: PreservedSpecimen
- **Type status:** Other material. **Occurrence:** recordedBy: Sanghyo Park; individualCount: 1; sex: male; lifeStage: adult; occurrenceID: E117DE82-F80B-5011-81E4-6DFB7C0EE280; **Taxon:** scientificName: Tropidocephalanigra; kingdom: Animal; phylum: Arthropoda; class: Insecta; order: Hemiptera; family: Delphacidae; genus: Tropidocephala ; **Location:** country: South Korea; stateProvince: Gyeongsangnam-do; municipality: Jinju-si; locality: 883, Oksan-ri, Munsan-eup; georeferenceProtocol: label; **Identification:** identifiedBy: Sanghyo Park; dateIdentified: 2023; **Event:** eventDate: 07-25-2023; **Record Level:** language: en; collectionCode: Insects; basisOfRecord: PreservedSpecimen
- **Type status:** Other material. **Occurrence:** recordedBy: Sanghyo Park; individualCount: 1; sex: female; lifeStage: adult; occurrenceID: 3F49C7BF-0677-58B9-B841-068FDE43D0EE; **Taxon:** scientificName: Tropidocephalanigra; kingdom: Animal; phylum: Arthropoda; class: Insecta; order: Hemiptera; family: Delphacidae; genus: Tropidocephala ; **Location:** country: South Korea; stateProvince: Gyeongsangnam-do; municipality: Jinju-si; locality: 883, Oksan-ri, Munsan-eup; georeferenceProtocol: label; **Identification:** identifiedBy: Sanghyo Park; dateIdentified: 2023; **Event:** eventDate: 07-25-2023; **Record Level:** language: en; collectionCode: Insects; basisOfRecord: PreservedSpecimen
- **Type status:** Other material. **Occurrence:** recordedBy: Sanghyo Park; individualCount: 4; sex: male; lifeStage: adult; occurrenceID: 311CAF51-31AD-5B6A-AA3D-17B20D959B61; **Taxon:** scientificName: Tropidocephalanigra; kingdom: Animal; phylum: Arthropoda; class: Insecta; order: Hemiptera; family: Delphacidae; genus: Tropidocephala ; **Location:** country: South Korea; stateProvince: Jeollanam-do; municipality: Suncheon-si; locality: 13-36, Yulchonsandan 5-ro, Haeryong-myeon; georeferenceProtocol: label; **Identification:** identifiedBy: Sanghyo Park; dateIdentified: 2022; **Event:** eventDate: 07-27-2022; **Record Level:** language: en; collectionCode: Insects; basisOfRecord: PreservedSpecimen
- **Type status:** Other material. **Occurrence:** recordedBy: Sanghyo Park; individualCount: 4; sex: female; lifeStage: adult; occurrenceID: 30A4984C-FB92-55EB-B0CB-44EE2D96EAA1; **Taxon:** scientificName: Tropidocephalanigra; kingdom: Animal; phylum: Arthropoda; class: Insecta; order: Hemiptera; family: Delphacidae; genus: Tropidocephala ; **Location:** country: South Korea; stateProvince: Jeollanam-do; municipality: Suncheon-si; locality: 13-36, Yulchonsandan 5-ro, Haeryong-myeon; georeferenceProtocol: label; **Identification:** identifiedBy: Sanghyo Park; dateIdentified: 2022; **Event:** eventDate: 07-27-2022; **Record Level:** language: en; collectionCode: Insects; basisOfRecord: PreservedSpecimen
- **Type status:** Other material. **Occurrence:** recordedBy: Sanghyo Park; individualCount: 3; sex: male; lifeStage: adult; occurrenceID: 277C7E88-3E9C-5A00-B009-E95B84B6B1B4; **Taxon:** scientificName: Tropidocephalanigra; kingdom: Animal; phylum: Arthropoda; class: Insecta; order: Hemiptera; family: Delphacidae; genus: Tropidocephala ; **Location:** country: South Korea; stateProvince: Jeollanam-do; municipality: Gwangyang-si; locality: 18-42, Hangman 8-ro; georeferenceProtocol: label; **Identification:** identifiedBy: Sanghyo Park; dateIdentified: 2022; **Event:** eventDate: 07-27-2022; **Record Level:** language: en; collectionCode: Insects; basisOfRecord: PreservedSpecimen
- **Type status:** Other material. **Occurrence:** recordedBy: Sanghyo Park; individualCount: 2; sex: male; lifeStage: adult; occurrenceID: 224DC3E9-76C8-5B22-A6F8-206D66F0FC48; **Taxon:** scientificName: Tropidocephalanigra; kingdom: Animal; phylum: Arthropoda; class: Insecta; order: Hemiptera; family: Delphacidae; genus: Tropidocephala ; **Location:** country: South Korea; stateProvince: Jeollanam-do; municipality: Goheung-gun; locality: 1892-67, Goheung-ro, Goheung-eup; georeferenceProtocol: label; **Identification:** identifiedBy: Sanghyo Park; dateIdentified: 2022; **Event:** eventDate: 03-30-2022; **Record Level:** language: en; collectionCode: Insects; basisOfRecord: PreservedSpecimen
- **Type status:** Other material. **Occurrence:** recordedBy: Sanghyo Park; individualCount: 2; sex: female; lifeStage: adult; occurrenceID: EBD79C59-1BC6-51A5-B3F0-071C642169D5; **Taxon:** scientificName: Tropidocephalanigra; kingdom: Animal; phylum: Arthropoda; class: Insecta; order: Hemiptera; family: Delphacidae; genus: Tropidocephala ; **Location:** country: South Korea; stateProvince: Jeju-do; municipality: Seogwipo-si; locality: 47, Wimihaean-ro, Namwon-eup; georeferenceProtocol: label; **Identification:** identifiedBy: Sanghyo Park; dateIdentified: 2023; **Event:** eventDate: 07-05-2023; **Record Level:** language: en; collectionCode: Insects; basisOfRecord: PreservedSpecimen
- **Type status:** Other material. **Occurrence:** recordedBy: Sanghyo Park; individualCount: 2; sex: male; lifeStage: adult; occurrenceID: D024C974-338A-5AD3-B83A-449A04A7702A; **Taxon:** scientificName: Tropidocephalanigra; kingdom: Animal; phylum: Arthropoda; class: Insecta; order: Hemiptera; family: Delphacidae; genus: Tropidocephala ; **Location:** country: South Korea; stateProvince: Jeju-do; municipality: Seogwipo-si; locality: 47, Wimihaean-ro, Namwon-eup; georeferenceProtocol: label; **Identification:** identifiedBy: Sanghyo Park; dateIdentified: 2023; **Event:** eventDate: 07-05-2023; **Record Level:** language: en; collectionCode: Insects; basisOfRecord: PreservedSpecimen

#### Description

Body length of male 3.8 mm. In male, body mostly brownish-black or black, except lateral carinae and frons which are yellow. Vertex of length twice the basal width, lateral convergent anteriorly, lateral carinae yellow. Mediolateral carinae mostly black, except base and apex which are yellow. Frons yellow at the lower part, blackish at the upper part, genae below eyes brownish-black to yellow towards frons. Clypeus brown with brownish-black carinae. Pronotum and mesonotum tricarinate with yellow carinae. Fore-wings mostly black, distinctly tinged transparent spot at apex. Abdominal segments dark brown (Figs 1d, 2d).

**Male genitalia.** Pygofer ovoid and posterior view with opening longer than broad. Genital styles slender and waved, subglobose on the apex. Aedeagus two branched slender needle-shaped (Figs 3d, 4d).

**Measurements.** Male macropterous form (n = 3). Body length without tegmina: 2.24 mm; body length with tegmina: 3.81 mm; body width: 0.86 mm; head length: 0.71 mm; head width (including eyes): 0.51 mm; 1^st^ antennal segment length: 0.07 mm; 2^nd^ antennal segment length: 0.11 mm; vertex length: 0.57 mm; vertex width: 0.30 mm; frons length: 0.71 mm; frons width: 0.32 mm; pronotum length: 0.24 mm; pronotum width: 0.67 mm; mesonotum length: 0.56 mm; mesonotum width: 0.59 mm.

#### Distribution

Korea, Japan, China (Anhui, Zhejiang) (Ding 2006).

#### Host plants

*Miscanthussinensis* Anderss (Poaceae) (in this study), *Imperatacylindrica* (Linn.) (Poaceae) (Ding 2006).

## Identification Keys

### Identification keys to the species of the tribe Tropidocephalini from Korea

**Table:** 

1	Vertex very short, width at base two times longer than length of vertex	2
–	Vertex longer than width at base	3
2	Genital styles strongly outwardly curved	*Epeurysadistincta*
–	Genital styles relatively straight, globose at apex	*Epeurysanawaii*
3	Vertex, pronotum and scutellum mostly yellowish-green	*Tropidocephalabrunnipennis*
–	Vertex, pronotum, scutellum and fore-wings dark brown to black	*Tropidocephalanigra*

## Supplementary Material

XML Treatment for Epeurysa distincta

XML Treatment for Epeurysa nawaii

XML Treatment for Tropidocephala brunnipennis

XML Treatment for Tropidocephala nigra

## Figures and Tables

**Figure 1a.**
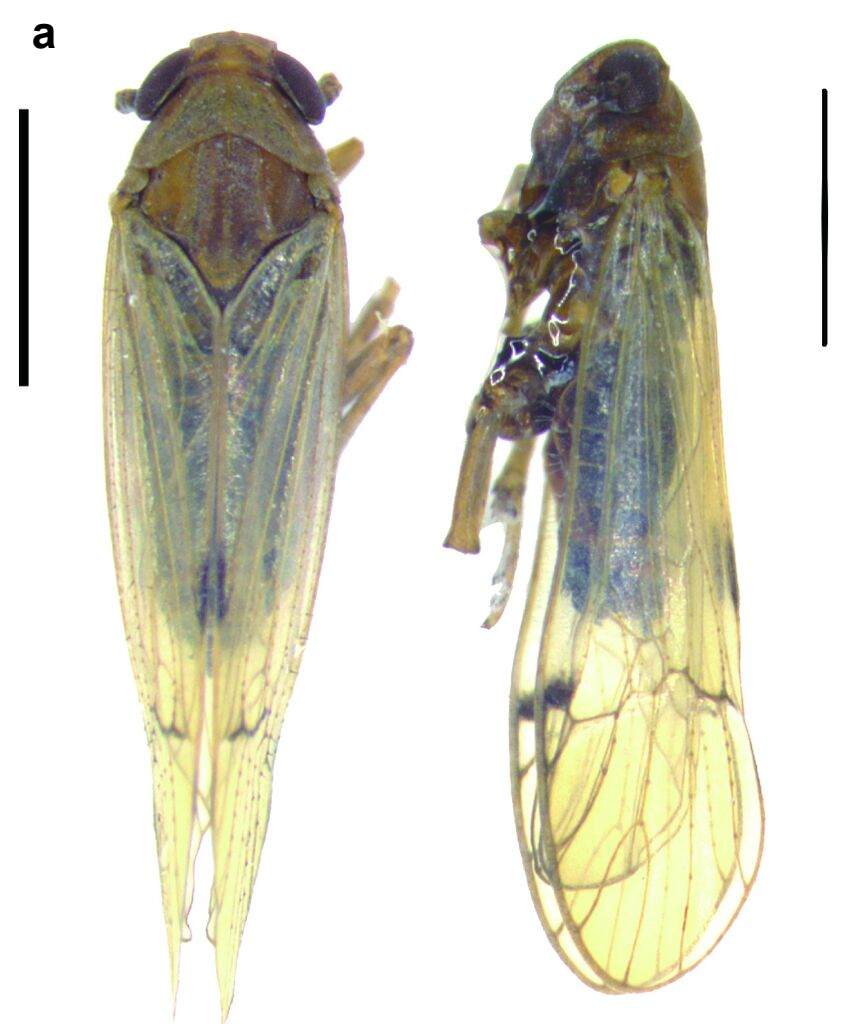
*Epeurysadistincta*, dorsal and lateral view;

**Figure 1b.**
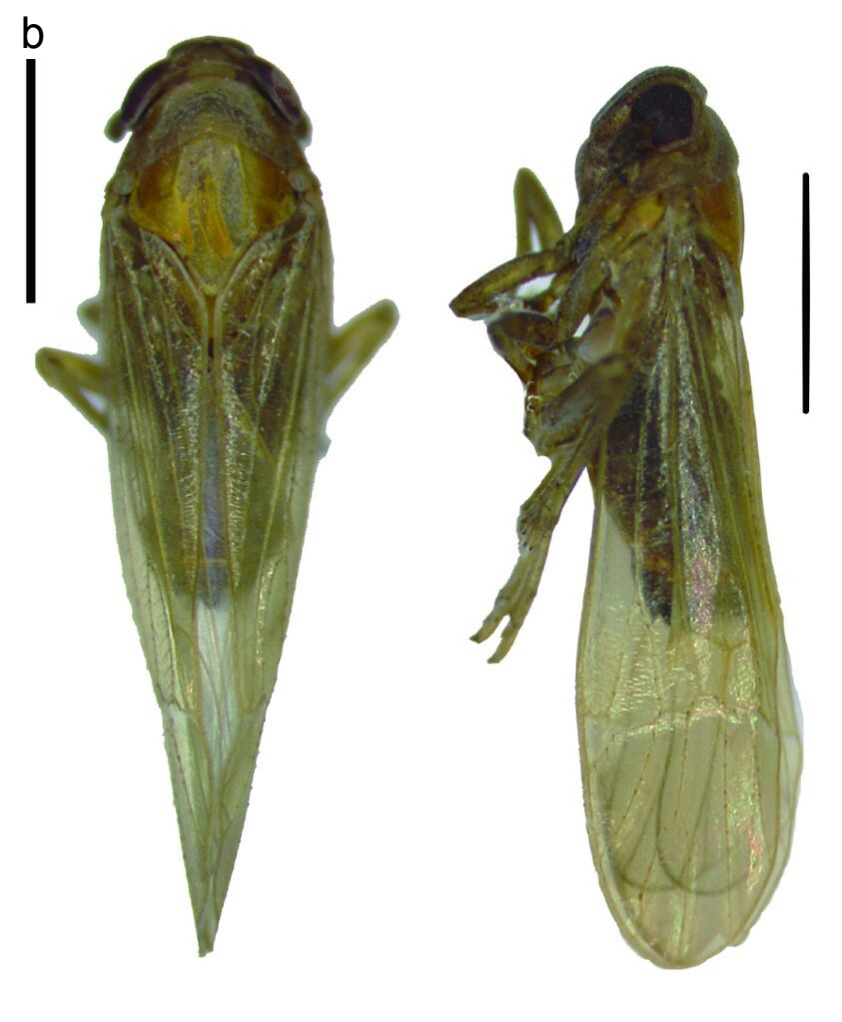
*Epeurysanawaii*, dorsal and lateral view;

**Figure 1c.**
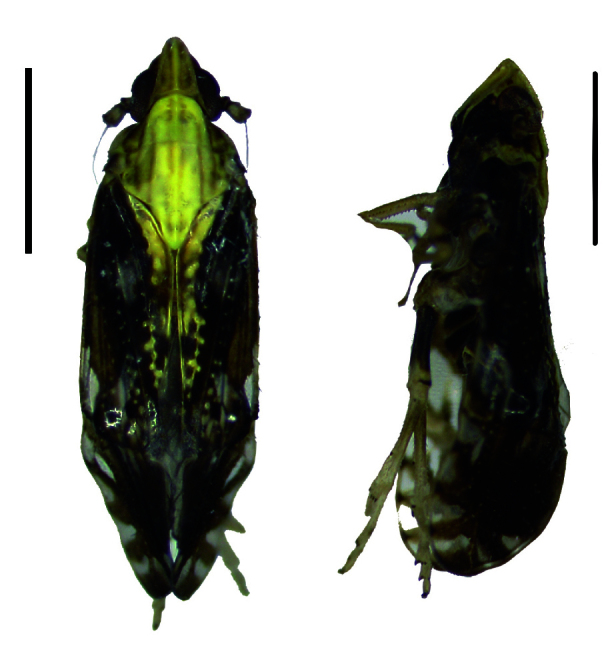
*Tropidocephalabrunnipennis*, dorsal and lateral view;

**Figure 1d.**
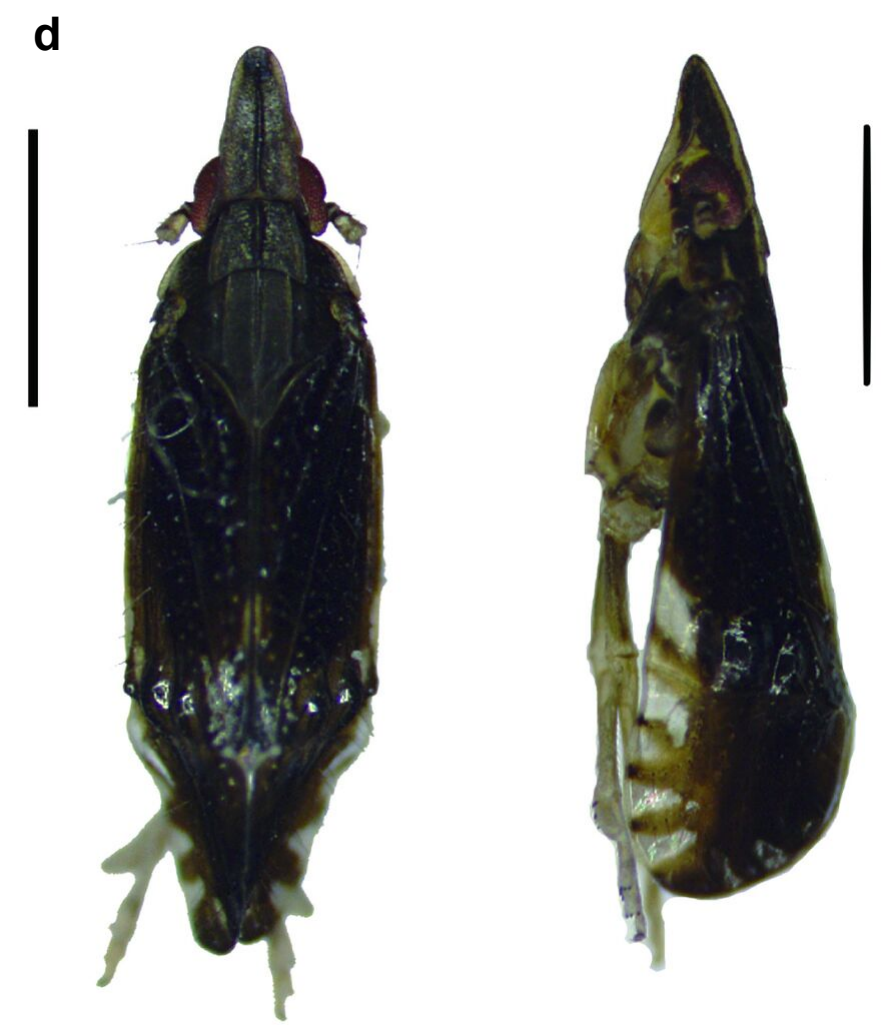
*Tropidocephalanigra*, dorsal and lateral view.

**Figure 2a.**
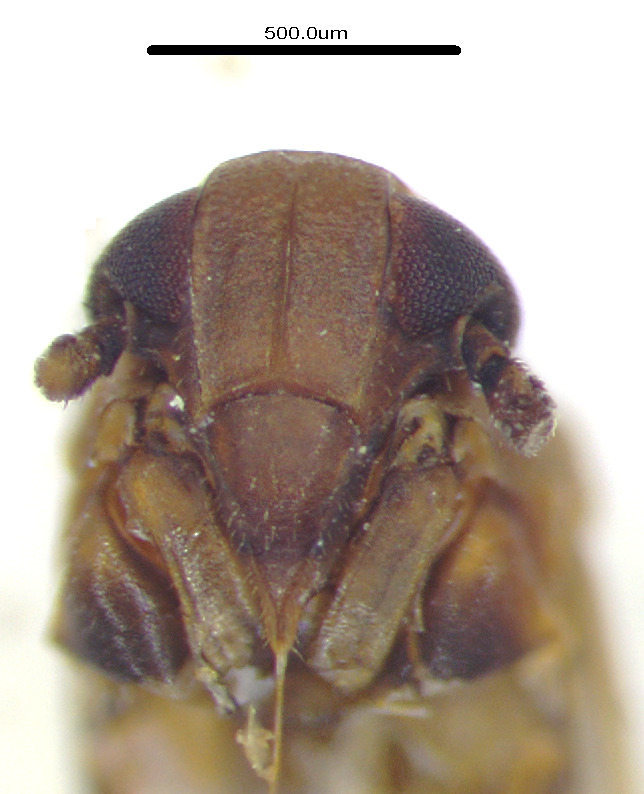
*Epeurysadistincta*, face;

**Figure 2b.**
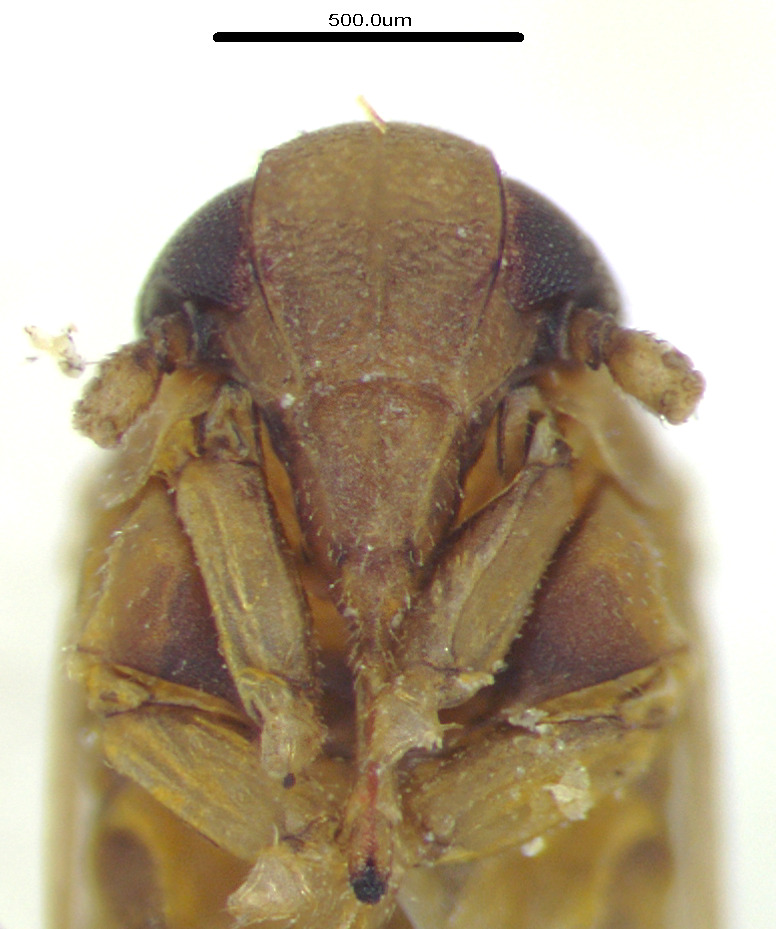
*Epeurysanawaii*, face;

**Figure 2c.**
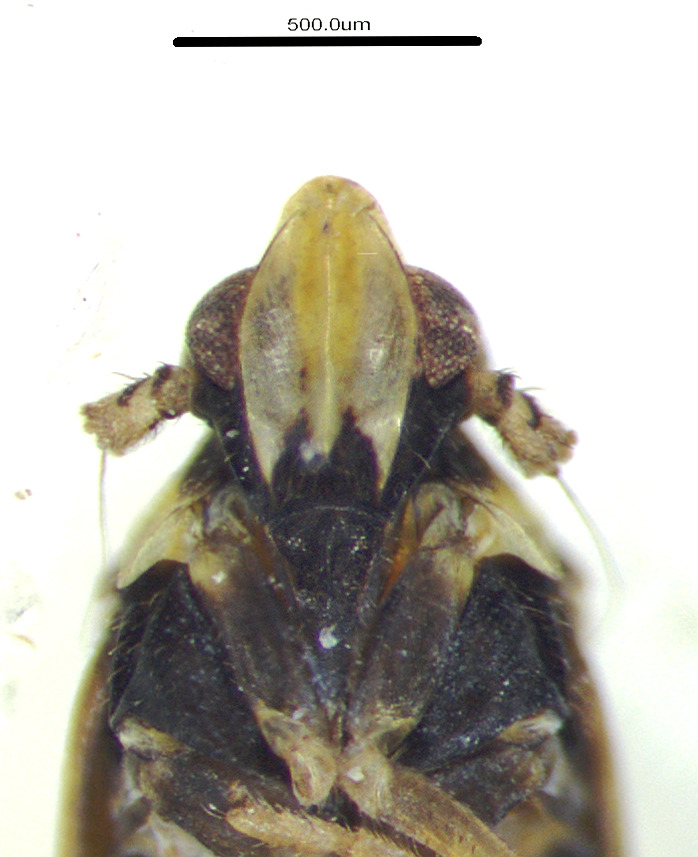
*Tropidocephalabrunnipennis*, face;

**Figure 2d.**
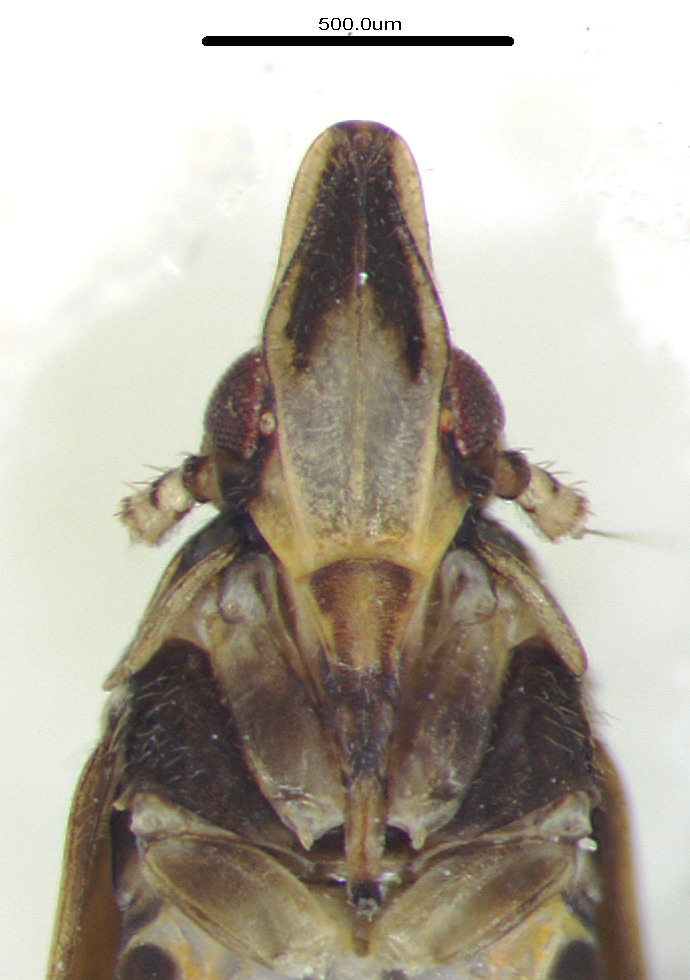
*Tropidocephalanigra*, face.

**Figure 3a.**
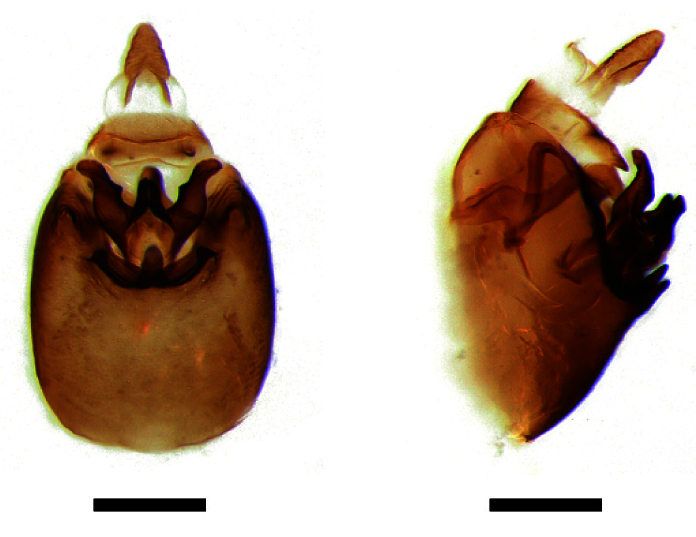
*Epeurysadistincta*, ventral view;

**Figure 3b.**
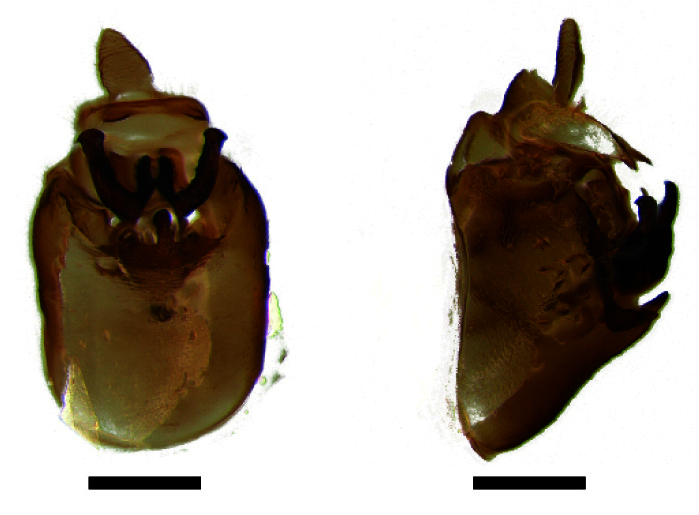
*Epeurysanawaii*, ventral view;

**Figure 3c.**
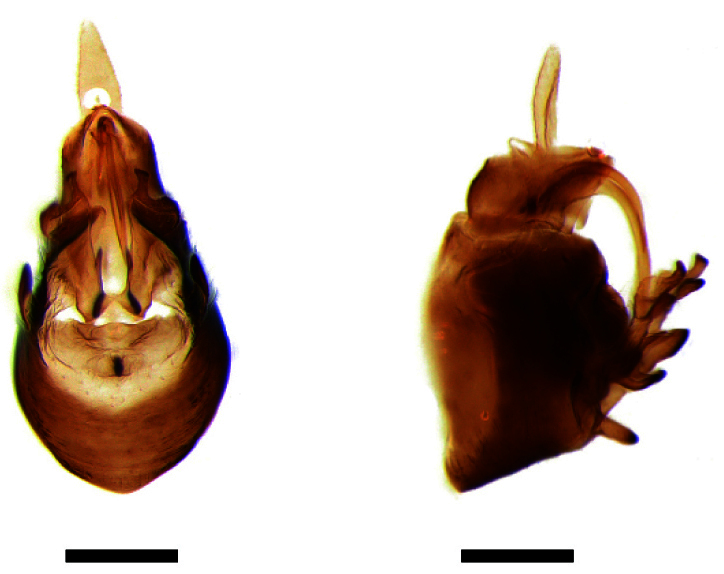
*Tropidocephalabrunnipennis*, ventral view;

**Figure 3d.**
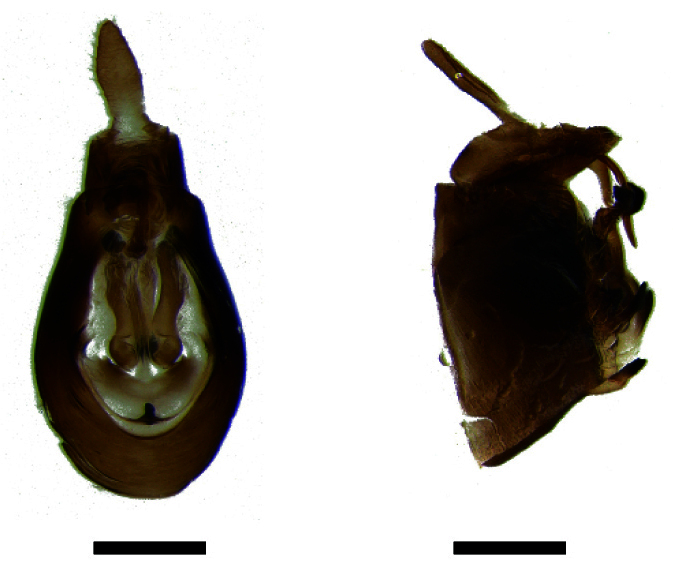
*Tropidocephalanigra*, ventral view.

**Figure 4a.**
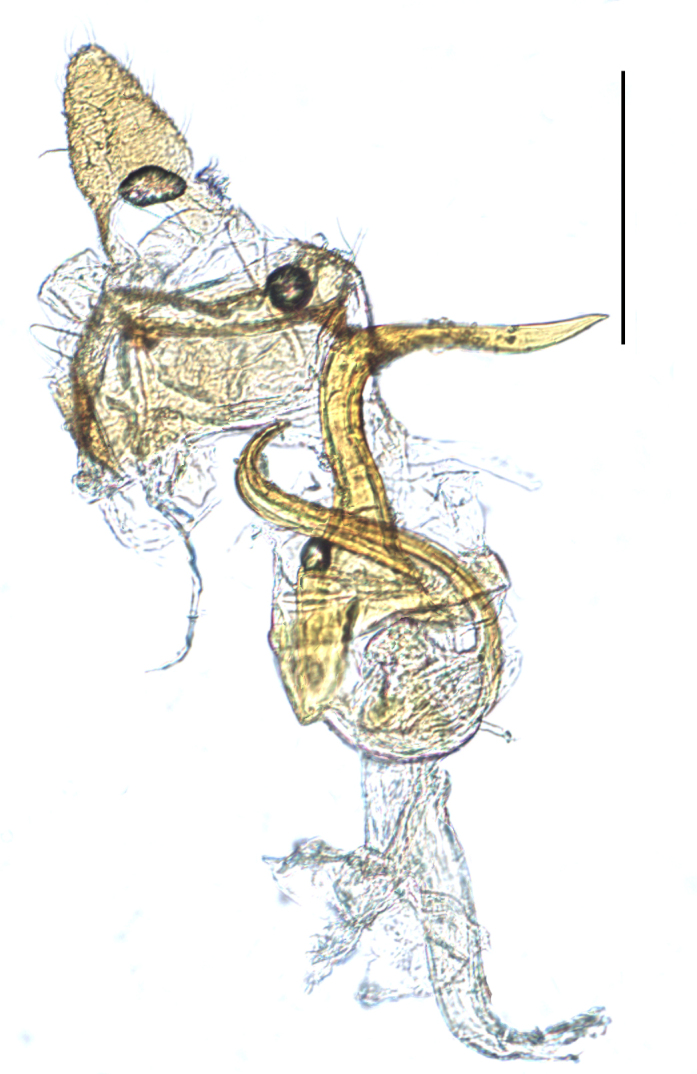
*Epeurysadistincta*, lateral view;

**Figure 4b.**
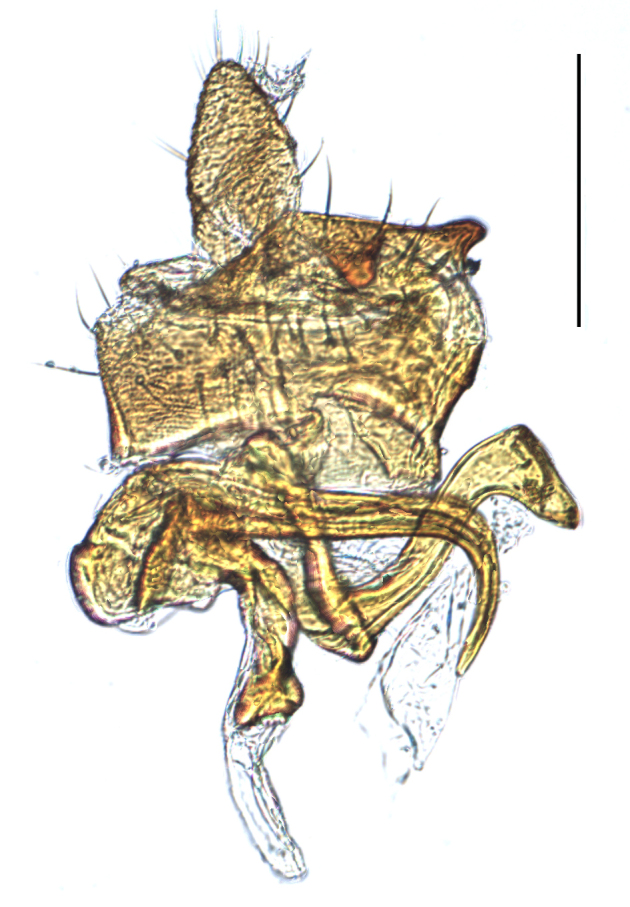
*Epeurysanawaii*, lateral view;

**Figure 4c.**
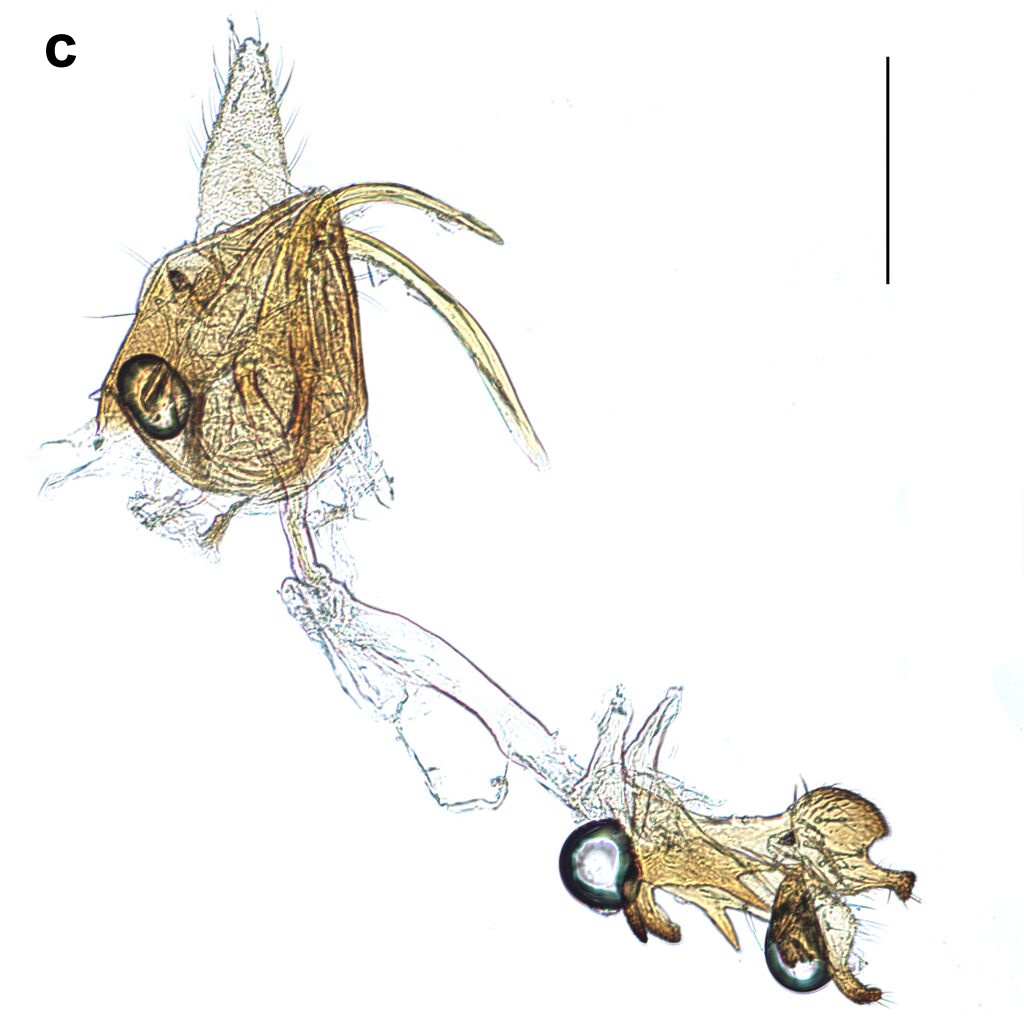
*Tropidocephalabrunnipennis*, lateral view;

**Figure 4d.**
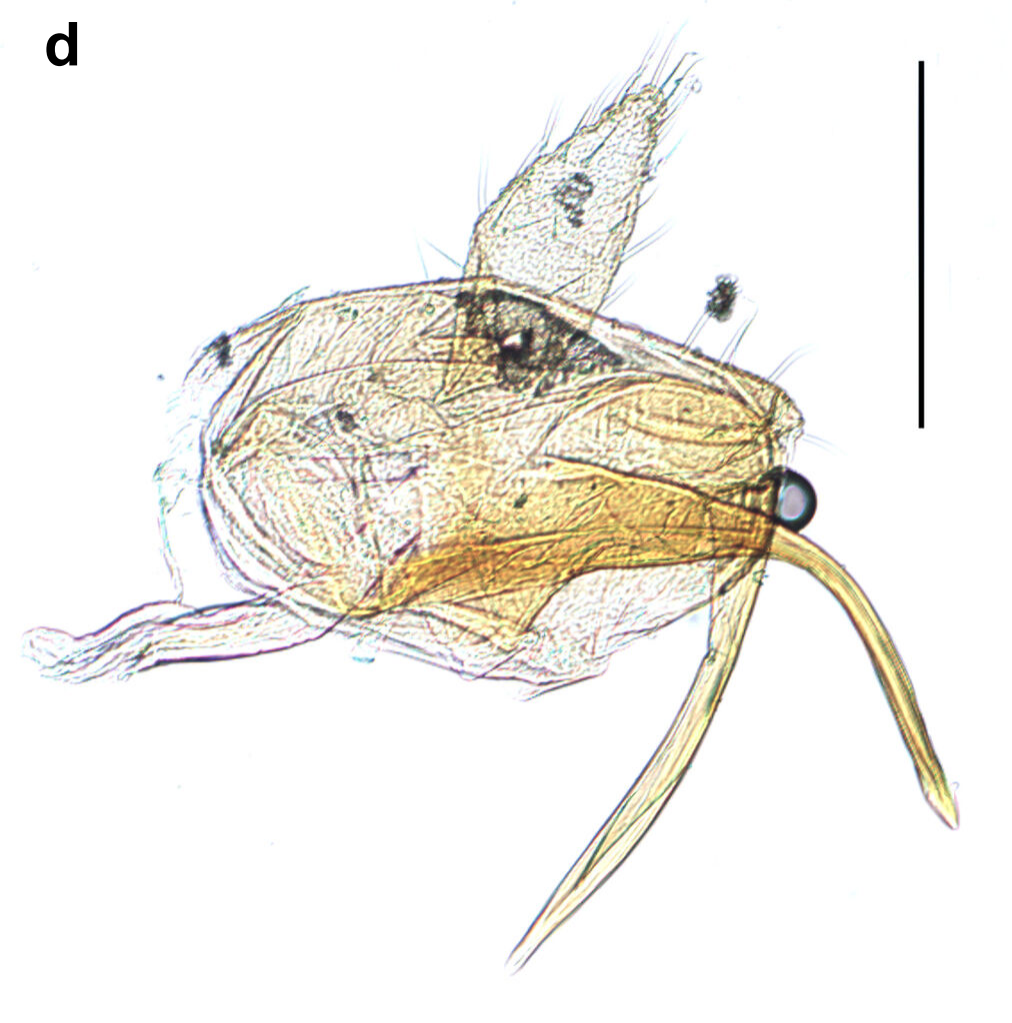
*Tropidocephalanigra*, lateral view.

**Figure 5a.**
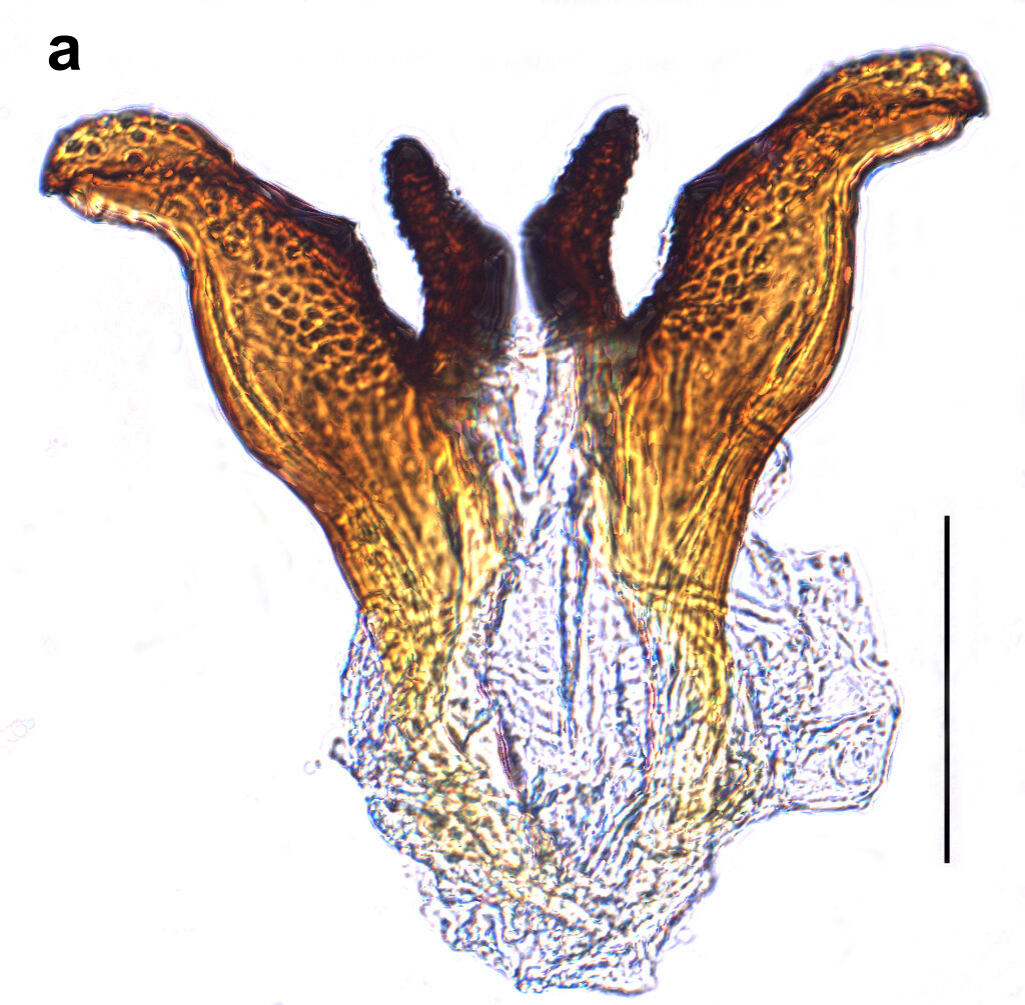
*Epeurysadistincta*, caudal view;

**Figure 5b.**
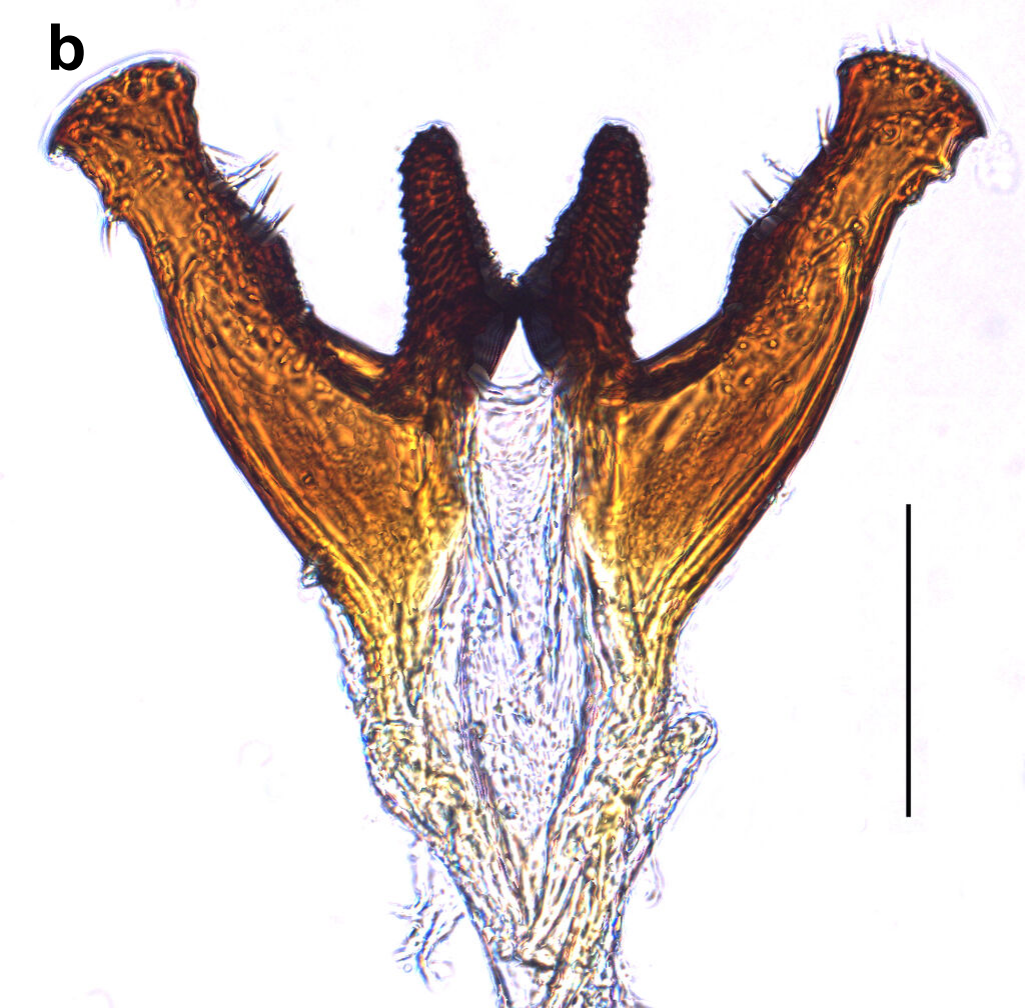
*Epeurysanawaii*, caudal view.
